# Isolated cardiophrenic angle node metastasis from ovarian primary. report of two cases

**DOI:** 10.1186/1749-8090-6-1

**Published:** 2011-01-05

**Authors:** Mark Ragusa, Jacopo Vannucci, Rosanna Capozzi, Niccolò Daddi, Nicola Avenia, Francesco Puma

**Affiliations:** 1 Thoracic Surgery Unit. University of Perugia Medical School. Ospedale S. Maria della Misericordia Perugia, Italy; 2 Endocrine and Soft Tissue of the Neck Surgery Unit. University of Perugia Medical School. Ospedale S. Maria Terni, Italy

## Abstract

Ovarian cancer is the most lethal gynaecologic malignancy. It usually spreads out of the abdomen involving thoraco-abdominal organs and serosal surface. This disease is poorly curable and surgery, at early stage, is supposed to achieve the best survival outcome. In systemic dissemination, chemiotherapy is indicated, sometimes with neoadjuvant aim. The most common clinical expressions of advanced ovarian carcinoma are multiple adenopathy, neoplastic pleuritis, peritoneal seeding and distant metastasis, mainly hepatic and pulmonary. Isolated adenopathy of the mediastinum is rare and isolated bilateral have never been described before. We report two cases of isolated bilateral cardiophrenic angle lymphnode metastasis from ovarian carcinoma, without peritoneal and pleural involvement. Both patients were successfully resected through minimally invasive thoracic surgery. About the role of surgery, few data are available but survival seems to be longer after resection thus, more investigation is required to make the indication to surgery more appropriate in advanced cases.

## Background

The cardiophrenic angle lymphnodes (CPLN) were classified by Rouviere into two groups: anterior prepericardiac and middle latero-pericardiac. The afferent lymphatics of CPLN drain areas from the diaphragm, liver, pleura and anterior abdominal wall and they empty into the internal mammary chain. Malignant lymphoma and metastases of abdominal or thoracic neoplasms have been mentioned to be possible causes of CPLN enlargement. Most of the times the disease is unilateral.

CPLN involvement may represent a staging and prognostic indicator for ovarian cancer [1]. Natural history of ovarian cancer entails extensive tumor dissemination on the peritoneal and pleural surface, with possible intrathoracic lymphnodes metastasis.

In the present paper we report two patients with isolated bilateral CPLN metastasis from previously resected ovarian carcinoma, with no peritoneal and pleural involvement.

### Case 1

**Figure 1:**
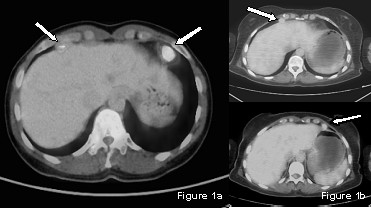
**PET-CT appearance of bilateral cardiophrenic angle node metastasis in the two cases reported**.

A 50-year-old woman was referred to our service for bilateral cardiophrenic angle mass. Two months earlier, the patient had undergone laparoscopic left ovariectomy with incidental diagnosis of cancer. Postoperative CA-125 value was within the normal range. Thoraco-abdominal computed tomography (CT) scan revealed bilateral neoplasms in the cardiophrenic angles, 2.5 and 1.5 cm in diameter. Fluorine 18-fluoro-2-deoxy-glucose-positron emission tomography (FDG-PET) scan evidenced enhanced uptake in the above mentioned sites (Figure 1a). The case was discussed at the multidisciplinary oncology round and indication for surgery was established. Videothoracoscopic complete removal of a capsulated yellowish cardiophrenic tumor was performed bilaterally. Pathology disclosed metastatic node colonization by papillary ovarian cancer in both specimens. The patient had an uneventful recovery and was discharged four days after the procedure. Two weeks later she underwent chemotherapy.

### Case 2

A 50-year-old woman, was admitted to our Hospital for bilateral cardiophrenic angle tumor. The patient had been submitted to laparotomic hysterectomy with bilateral salpingo-oophorectomy two years earlier, for ovarian papillary serous adenocarcinoma (pT1bN0Mx). Preoperative Ca-125 value was 14.00 U/ml. Three cycles of adjuvant chemotherapy with carboplatin and taxol were administered. During follow-up, FDG-PET scan revealed an increased uptake in a bilateral 1.5 cm cardiophrenic tumor, not recognized on CT scan (Figure 1b). After discussion with the referring oncologist and the patient, she underwent a sequential bilateral videothoracoscopy with complete cardiophrenic tumor removal. Pathology disclosed metastatic node colonization by papillary ovarian cancer in both specimens. After an uneventful recovery, the patient was discharged five days after surgery and returned to the oncologist for chemotherapy.

## Conclusion

Ovarian carcinoma remains the most lethal gynaecologic malignancy. It usually spreads out of the abdomen along different routes: lymphatic, haematogenous and transcaelomic. One of its hallmarks is the possible peritoneal and pleural dissemination. Mediastinal lymphnode metastasis (stage IV) entails a definite worsening of prognosis [2]. CPLN colonization is frequently associated with extensive intrathoracic disease, typically represented by right-sided pleural effusion [1]. Such behaviour is explained by the anatomic arrangement of abdominal lymphatic drainage, which follows a clockwise route, involving first the thoracic lymphatic stations on the right side.

Isolated bilateral metastasis is to be considered anecdotical and, to our knowledge, bilateral involvement without pleural effusion was never reported in the English Literature. In our patients FDG-PET scan facilitated identification and proper diagnosis of CPLN metastasis. In one patient CT scan did not clearly demonstrate nodal disease. The possible anatomical pathway for tumor spread in the cases herein reported is an unanswered question, considering that serosal surfaces and intra-abdominal viscera were apparently unaffected by disease.

An interesting, potentially misleading, feature of metastatic supradiaphragmatic nodes from ovarian primary, is calcification. Although not observed in our cases, such aspect is reported with an incidence up to 35%, and must not be overlooked. Calcified intrathoracic nodes in patients with previous ovarian serous adenocarcinoma cannot be ruled out as granulomatous disease, but metastatic deposits must be excluded. A hint to the latter hypothesis is the progressive growth of the involved station [3], also considering that, in such circumstances, FDG-PET scan is not entirely reliable because granulomatous lymphadenitis as well may show an increased FDG-uptake.

Surgery is carried out in order to achieve histologic diagnosis, disease staging, and prolonged survival. Videothoracoscopy is specifically fit for such procedure, as recently stated by Lim et Al.[1]. The minimally invasive approach enables thorough exploration of the entire pleural cavity, easy resection of even small nodes deeply sited within the pericardial fat, and the one-stage removal of bilateral CPLN growths. Resection of isolated node metastases can improve outlook, particularly for slow growing tumors. In such setting, progression-free survival before relapse does not appear to be a reliable indicator of prognosis, as it is for many other cancers. Tumor growth rate seems a more sound parameter [2]. Treatment of recurrent epithelial ovarian cancer is based on various considerations: recurrence site, general conditions of the patient, disease-free interval (with the above-mentioned caveat), growth rate, response to first-line chemotherapy.

In presence of isolated CPLN relapse, the patient may be included in the Isolated Lymph Node Relapse group, a subset appearing to gain from surgery in terms of survival [4]. On the other hand, only one series of video-assisted transthoracic resection of lymph node and pleural metastasis from ovarian cancer is available, therefore further data are required to clarify the role of surgery in downstaging ovarian cancer diffusion to the mediastinum and thoracic cavity [1].

Written informed consent was obtained from the patients for publication of this case report and any accompanying images. A copy of the written consent is available for review by the Editor-in-chief of this journal.

## List of abbreviations

CPLN: cardiophrenic angle lymphnodes; CT: computed tomography; FDG-PET: Fluorine 18-fluoro-2-deoxy-glucose-positron emission tomography;

## Competing interests

The authors declare that they have no competing interests.

## Authors' contributions

MR, JV and FP wrote the article, RC and ND collected the clinical information and selected the images, MR and NA analyzed the English Literature. FP drafted the final manuscript. All authors approved the final manuscript to be published.
